# A Manual of Surgical Treatment

**Published:** 1914-06

**Authors:** 


**A Manual of Surgical Treatment.** By Sir WATSON CHEYNE, Bart., C.B., F.R.C.S., and F. F. BURGHARD, M.S., F.R.C.S. New edition, entirely revised, and largely re-written with the assistance of T. P. LEGG, M.S., F.R.C.S., and ARTHUR EDMUNDS, M.S., F.R.C.S. Vol. V. Pp. xxviii, 619. London: Longmans, Green & Co. 1913. Price, 21s. net.

This volume, which completes the revised edition of this manual, deals with the surgical affections of the pancreas, liver, spleen, neck, thyroid, larynx, breast, thorax, lungs, heart, and genito-urinary organs. The question of choice between cholecystectomy and cholecystostomy is left somewhat open, the former being chiefly recommended for cases with diseased gall-bladder and a common duct free from obstruction. In the section on the spleen there is no very clear account of Banti's disease, with the indications as to splenectomy in its various stages. In the treatment of spasmodic torticollis it is not mentioned that usually the posterior cervical nerves to be resected are those on the opposite side to that of the affected sterno-mastoid.

The surgical treatment of exophthalmic goitre is put forward, and its indications discussed in a clear manner.

In cancer of the breast much importance is attached to a wide removal of skin, and the incision is to be planned according to the situation of the tumour in the breast.

In the surgery of the thorax full illustrated descriptions are given of the various differential pressure chambers, but it is unfortunate that in mentioning the intratracheal anæsthesia as a substitute for these, the least efficient apparatus for this purpose is mentioned, viz. Boyle's, in which the foot bellows never gives a constant pressure.

In diseases of the prostate the perineal route for removal of the gland is said to be indicated in the fibrous and hard conditions of the gland.

There is a very good account of the operative treatment of ectopia vesicæ, the trigone being implanted into the pelvic colon.

A chapter on the modern methods of cystoscopy, pyelography and estimation of the functional conditions of the kidneys is written by Thomson Walker, and is a valuable addition to the work.

In the article on nephropexy is included a good account of the anterior operation which is recommended because of the accuracy with which stitching can be done, and the facility that is afforded by the anterior incision of making exploration of the abdomen.

The authors of this work may be congratulated on the revised edition, which once more makes this the standard English work on surgical treatment.

